# Phyto Synthesis of Manganese-Doped Zinc Nanoparticles Using *Carica papaya* Leaves: Structural Properties and Its Evaluation for Catalytic, Antibacterial and Antioxidant Activities

**DOI:** 10.3390/polym14091827

**Published:** 2022-04-29

**Authors:** Mir Waqas Alam, Hassan S. Al Qahtani, Muhammad Aamir, Alaaedeen Abuzir, Muhammad Shuaib Khan, Maryam Albuhulayqah, Shehla Mushtaq, Noushi Zaidi, Ambikapathi Ramya

**Affiliations:** 1 Al Bilad Bank Scholarly Chair for Food Security in Saudi Arabia, The Deanship of Scientific Research, The Vice Presidency for Graduate Studies and Scientific Research, King Faisal University, Al-Ahsa 31982, Saudi Arabia; msadiq@kfu.edu.sa (M.A.); aabuzir@kfu.edu.sa (A.A.); 218008189@student.kfu.edu.sa (M.A.); nzaidi@kfu.edu.sa (N.Z.); 2 Department of Physics, College of Science, King Faisal University, Al-Ahsa 31982, Saudi Arabia; 3 EXPEC Advanced Research Centre, Saudi Aramco, Dhahran 31311, Saudi Arabia; hassan.alqahtani.2@aramco.com; 4 Department of Basic Science, Preparatory Year Deanship, King Faisal University, Al-Ahsa 31982, Saudi Arabia; 5 International Research Center for Renewable Energy (IRCRE), State Key Laboratory of Multiphase Flow in Power Engineering (MPFE), Xi’an Jiaotong University, 28 West Xianning Road, Xi’an 710049, China; m.shuaibkhan@mail.xjtu.edu.cn; 6 Department of Biomedical Engineering, College of Engineering, King Faisal University, Al-Ahsa 31982, Saudi Arabia; 7 School of Natural Sciences, National University of Sciences & Technology, Islamabad 44000, Pakistan; shehla.mushtaq@sns.nust.edu.pk; 8 Department of Agriculture Engineering, Rathinam Technical Campus, Coimbatore 641021, Tamilnadu, India; ramya.agri@rathinam.in

**Keywords:** degradation, low cost, ecofriendly, antioxidant, *p*-nitrophenol reduction, hydrodynamic diameter

## Abstract

The current study aims to synthesize bimetal oxide nanoparticles (zinc and manganese ions) using the *carica papaya* leaf extract. The crystallite size of the nanoparticle from X-ray diffraction method was found to be 19.23 nm. The nanosheet morphology was established from Scanning Electron Microscopy. Energy-dispersive X-ray diffraction was used to determine the elemental content of the synthesized material. The atomic percentage of Mn and Zn was found to be 15.13 and 26.63. The weight percentage of Mn and Zn was found to be 7.08 and 10.40. From dynamic light scattering analysis, the hydrodynamic diameter and zeta potential was found to be 135.1 nm and −33.36 eV. The 1,1-diphenyl-2-picryl hydroxyl radical, hydroxyl radical, FRAP, and hydrogen peroxide scavenging tests were used to investigate the antioxidant activity of Mn-Zn NPs. Mn-Zn NPs have substantial antioxidant properties. The photocatalytic activity of the Mn-Zn NPs was assessed by their ability to degrade Erichrome black T (87.67%), methyl red dye (78.54%), and methyl orange dye (69.79%). Additionally, it had significant antimicrobial action *S. typhi* showed a higher zone of inhibition 14.3 ± 0.64 mm. Mn-Zn nanoparticles were utilized as a catalyst for *p*-nitrophenol reduction. The bimetal oxide Mn-Zn NPs synthesized using *C. papaya* leaf extract exhibited promising dye degradation activity in wastewater treatment. Thus, the aforementioned approach will be a novel, low cost and ecofriendly approach.

## 1. Introduction

Dyes are the major industrial pollutants liberated in water bodies and nitrophenols are believed to be common organic pollutants found in water produced from agriculture [1,2]. The people who consumed polluted water may experience headaches, nausea, etc., especially in children and pregnant women. The polluted water affects human beings and aquatic environment. It affects the photosynthetic activity of aquatic life and reduces diversity due to bioaccumulation. In addition to that agricultural irrigation is considered as one the largest global consumption of water where we use low quality or wastewater. Farmers mostly use the wastewater for irrigation purposes without conducting a risk assessment, which can pose a high risk to the quality of soil and human health. The common source of wastewater is industrial waste, where a large number of dyes have been found in textile waste waters, because of poor washing operation. When effluents are used for irrigation purposes worldwide, it contaminates the soil and hence causes several crop plants to become toxic. In addition, the dye 4-Nitrophenol (4-NP) is also found in water bodies and soils and is considered a key source of environmental impact and health risk [3]. Even lower concentrations are harmful to health and potentially carcinogenic and mutagenic. 

The removal of organic pollutants such as organic, textile dyes and 4-Nitrophenol (4-NP) is difficult, due to the high concentration of organic molecules and its complex composition. Numerous techniques have been utilized to remove the pollutants from wastewater, such as adsorption, electrocoagulation, microfiltration, flocculation, etc. However, these techniques are only used to separate the pollutants from affected water and transfer to other storage places such as sludge dumps, and it does not degrade the pollutants. An alternate method is required to overcome this problem [4,5]. 

In the current scenario, nanoscience and nanotechnology plays a significant role in various fields such as chemistry, physics, biology, materials science and engineering, etc. [6]. The transition metal/metal oxide nanoparticles are quite interesting because of their distinctive optical, thermal, magnetic and electrical properties [7]. The metals such as manganese (Mn), Zinc (Zn), etc., are the most significant metals in diverse fields due to their excellent physic-chemical properties and so a lot of researchers pay attention to these metals [8]. Recent studies have shown that bimetal oxide nanoparticles are more efficient than monometallic nanoparticles. Changes in electrical surface characteristics occur when metals are combined. In bimetal oxide nanostructures, the synergistic effect reveals novel chemical, thermal, catalytic, electrical, mechanical, and optical capabilities. Bimetal oxide nanoparticles displayed in a variety of shapes and sizes, as well as morphologies. The metal nanoparticles were used in environmental remediation, catalysis, sensors, transistors, optoelectronics, microelectronics, and biomedical application [9,10,11]. The heterogeneous catalysis provides an everlasting opportunity in the degradation/conversion of organic pollutants by reduction or oxidation reactions.

Due to its unique electronic, optical, chemical and catalytic properties, ZnO has been used in cutting-edge applications such as cosmetics, electronics, biology, and the medicinal industry [12,13]. However, due to the higher energy gap and absorption of pure ZnO, NP have several drawbacks in photocatalytic activity. Doping of transition metals such as Fe, Mn, and Ni is highly utilized to improve the energy gap for ZnO nanoparticles and to improve the usage of visible light photo-catalytic applications, [14]. Based on previous reports, its shown that the Mn have strong solubility with ZnO nanoparticles and their combination is proved to be very efficient for several electronic application [15,16].

Nanomaterials have been synthesized by physical, chemical, and green methods. Among them, the green method is an emerging technique due to being economic, environmentally friendly, easy to handle and less toxic [9,10]. Synthesis of nanoparticles using plant-based extracts as potential capping and reducing agents is often known as green synthesis. Plant materials such as leaves, roots, stems, seeds, etc. are usually used as bio-reductant in the synthesis of metallic nanoparticles rather than bacteria, yeast, fungi, and algae. Generally, phenolic compounds including alkaloids, flavonoids, amino acids, lipids, and carbohydrates existed in the plant materials. The plant materials can influence the properties of the synthesized nanoparticles by enhancing their functionality optimally for biomedical applications [17]. 

*Carica papaya (C. papaya)* belongs to the Caricaceae family, and its leaf extract has been used to increase the platelet count and is used as a medicine for treating dengue fever [18]. *C. papaya* leaf extract constitutes proteins and sugars as the primary constituent. In addition, phenols, flavonoids, and tannins are considered to be a secondary constituent [19]. To the best of our knowledge and from a critical literature survey, the phyto-synthesis of Mn-Zn nanofiller based on *C. papaya* by employing a low cost, ecofriendly approach has not been reported before. In the current study, photosynthesized bimetal oxide Mn-Zn nanoparticles are employed to improve the qualities of nanoparticles by modifying combustion and physical properties. Due to the efficient properties of bimetal oxide nanoparticles, the present study aims to design nanoparticles for environmental remediation issues. The prepared nanoparticles are considered as an efficient way to remove the pollutant from wastewater and for 4-NP reduction. The results show the prepared nanoparticles are high effective for several applications such as catalytic, antibacterial and antioxidant activities. 

## 2. Materials and Method

### 2.1. Chemicals and Reagents

The chemicals such as Manganese chloride tetrahydrate (MnCl_2_·4H_2_O), Zinc chloride tetrahydrate (ZnCl_2_·4H_2_O), phenyl hydrazine (C_6_H_5_NHNH_2_), *p*-nitrophenol (C_6_H_4_(NO_2_)OH), hydrogen peroxide (H_2_O_2_), sodium hydroxide (NaOH), and ethanol (C_2_H_5_OH) were purchased from Sigma Aldrich (St. Louis, MO, USA).

### 2.2. Collection and Preparation of C. papaya Leaves Extract

The *C. papaya* leaves were collected from the Coimbatore region, Tamil Nadu, India. The collected plant leaves were washed in running water followed by double distilled water to remove the dust particles. Then, it was chopped into small pieces and dried at room temperature (31 °C). The dried leaves were powdered using a grinding machine and stored in an air-tight container for further use. In 100 mL double distilled water, 1 g of *C. papaya* leaves were added and stirred at 60 °C, and the solution was reduced to 40 mL. The solution containing leaf extracts was filtered and utilized for nanoparticle synthesis.

### 2.3. Green Synthesis of C. papaya-Manganese–Zinc Nanoparticles (C. papaya-Mn-Zn NPs)

Briefly, 100 mL ZnCl_2_·4H_2_O (0.1 M) and 100 mL MnCl_2_·4H_2_O (0.1 M) solutions were mixed in a round bottom flask. To the above reaction mixture, 20 mL of *C. papaya* leaves extracts were added dropwise. Then, (0.2 M) NaOH solution was added dropwise to the mixture while frequently stirring until a basic pH was obtained. The reaction mixture was kept in the darkroom for up to 24 h and frequently monitored for the formation of *C. papaya*-Mn-Zn NPs. The formation of *C. papaya*-Mn-Zn NPs was confirmed by the color change from green to dark brown. Finally, the synthesized NPs were washed by centrifuging at 3000 rpm using water and ethanol mixture (3:1 ratio) to eliminate the impurities and unreacted precursors, dried at 60 °C to remove the moisture and foreign materials. *C. papaya*-Mn-Zn NPs were stored for characterization and application purposes. 

### 2.4. Analysis of Green Synthesized Mn-Zn Bimetal Oxide Nanoparticles

The *C. papaya*-Mn-Zn NPs were analyzed by UV-Vis Spectrophotometer (Shimadzu UV-2600 ISR 2600 PLUS spectrophotometer, Tokyo, Japan) to record the absorption spectra of the synthesized NPs. FT-IR analysis was carried out to identify the possible biomolecules responsible for reducing metal ions and capping of Mn-Zn NPs synthesized by *C. papaya*. The FT-IR spectrum was recorded by Shimadzu, Tokyo, Japan using KBr powder as a standard. The crystallite size and phase of the NP was analyzed by X-ray diffraction using Ultima IV-Rigaku diffractometer, Tokyo, Japan. The XRD measurement was performed with Cu Kα radiation (λ-1.540 Å, 40 kV, 25 mA) and it was recorded in the range of 10–90°. The morphology and average particle size of the *C. papaya*-Mn-Zn NPs were analyzed by Scanning Electron Microscopy using TESCAN MIRA3, Australia. The atomic and weight percentage of the NPs were found by using EDX analysis. The hydrodynamic diameter and zeta potential of *C. papaya*-Mn-Zn NPs were analyzed by Dynamic Light Scattering (DLS) using Horiba, SZ-100, Kyoto, Japan. The synthesized nanomaterials were diluted and dispersed by the sonication process for 20 min in an ultrasonic bath and were analyzed directly using DLS instrument and the temperature was set as 25 °C during the analysis. The graphite electrode was used to perform and measure the synthesized nanoparticles’ zeta potential, and the temperature was set as 25 °C during the analysis. 

### 2.5. Anti-Oxidant Activity

#### 2.5.1. DPPH

The antioxidant potency of *C. papaya*-Mn-Zn NPs was evaluated using 1, 1-diphenyl-2-picrylhydrazyl radical (DPPH) assay by adopting the protocols of Rajagopal et al. [20]. For this assay, 100 µL of *C. papaya*-Mn-Zn NPs was mixed with 100 µL of ethanol and 50 µL of DPPH solution and maintained in the dark for 30 min. The solution’s absorbance was measured at 595 nm.

#### 2.5.2. Hydrogen Peroxide (H_2_O_2_) Radical Scavenging Assay

In a hydrogen peroxide (40 mM) solution, 0.1 M phosphate-buffered saline was added. Then, 600 μL of hydrogen peroxide solution and *C. papaya*-Mn-Zn NPs containing samples of varying concentrations were added and kept for 10 min at room temperature. A UV-Vis spectrophotometer was used to measure the absorbance at 230 nm, which was then compared to a blank to get the overall absorbance (without hydrogen peroxide). The ascorbic acid was used as reducing agent as well as capping agent in the nanoparticle synthesis which makes the process non-toxic, economical and environmentally friendly [21]. The hydrogen peroxide scavenging percentage was calculated using Equation (1) [22].

H_2_O_2_ scavenging activity percentage= [(A_0_ − A_1_)/A_0_] × 100 (1)

where A_0_ = Absorbance of the control and A_1_ = Absorbance of the sample.

#### 2.5.3. Hydroxyl-Radical Scavenging Activity

The hydroxyl radical scavenging activity was performed using salicylic acid Xie et al. [23]. The synthesized *C. papaya*-Mn-Zn NPs was diluted in 1 mL of deionized water at various concentrations (200 to 1000 μg/mL). The reaction mixture contains 1 μL of *C. papaya*-Mn-Zn NPs in 1 μL of salicylic acid (9 mM), 1 μL of ferrous sulphate (9 mM), and 1 μL of hydrogen peroxide (9 mM). After incubation, the reaction mixture was incubated at 37 °C in a water bath for 60 min. The absorbance of the mixture was measured at 510 nm using a UV-Vis spectrophotometer. For this experiment, ascorbic acid without *C. papaya*-Mn-Zn NPs was used as a control. The experiment was performed in triplicate to confirm the hydroxyl scavenging properties, and the amount of hydroxyl generated was quantified.

#### 2.5.4. Ferric Reducing Antioxidant Power Assay

*C. papaya*-Mn-Zn NPs were evaluated in terms of antioxidant capacity using the method published by Khan et al. [24]. Corresponding concentration was determined as the antioxidant concentration, which increases the absorption of a 1 mM/L quantity of Fe in the ferric reduced antioxidant energy (FRAP) test. The equivalent amount of antioxidant was calculated to enhance the absorption of the FRAP test, equal to the expected absorbance value of a 1 mM/L *C. papaya*-Mn-Zn NPs solution concentration.

### 2.6. Antibacterial Activity

The bactericidal activity of *C. papaya*-Mn-Zn NPs in the Muller Hinton Agar (MHA) medium was determined using the Kirby–Bauer method [25]. MHA medium was sterilized on sterile Petri plates at 121 °C at 15 pounds for 20 min. Four bacterial strains including *Escherichia coli* and *Klebsiella pneumoniae* (Gram-negative), *Staphylococcus aureus* and *Salmonella typhi* (Gram-positive) were used to assess the antibacterial activity of synthesized *C. papaya*-Mn-Zn NPs. The bacterial strains were swabbed using a sterile cotton swab on the surface of the medium after completely solidifying and incubated for 15 min. For antibacterial activity, three different concentrations of (1 mg/mL, 2 mg/mL, and 3 mg/mL) stock solution of *C. papaya*-Mn-Zn NPs were prepared using 50% DMSO. Each concentration was added into three wells, each containing 100 µL. The zone of inhibition (ZOI) was measured after 24 h of incubation at 37 °C.

### 2.7. Photocatalytic Activity of C. papaya-Mn-Zn NPs 

The photocatalytic activity of *C. papaya*-Mn-Zn NPs were evaluated against methyl red (MR), Eriochrome black T (EBT), and methyl orange (MO) as a pollutant. The stock solution was prepared as 10 mg/L using double distilled water for each dye in different SMF. The photocatalytic degradation reaction was performed using the batch reactor system under solar irradiation with *C. papaya*-Mn-Zn NPs as a photocatalyst. The open rectangular tray was used as a reactor in the degradation processed reactor consists of 100 mL dye solution with known weight of (30, 60, 120, 180, and 240 mg) photocatalyst. The reaction mixture was stirred for 30 min under dark conditions to attain equilibrium before the degradation process started and the pH of the solution was maintained to be neutral. After that, the degradation process started under sunlight. The intensity of sunlight was measured every 30 min using a digital flux meter. The average intensity of sunlight irradiation was measured as approximately 840 lux. The intensity was nearly constant throughout the experiment. The stock solution of dyes was measured using UV-Vis spectrometer, which was found to be 425, 566, and 464 nm for MR, EBT, and MO, respectively. The degradation percentage of dye was calculated using Equation (2) [26].

(2) $$\mathrm{Degradation}\;\left(\%\right)\;\frac{A_{0}-A}{A_{0}}\times 100$$

whereas *A*_0_ is the initial concentration of dye solution and *A* is the concentration of dye solution after photocatalytic activity.

### 2.8. Conversion of p-Nitrophenol to p-Aminophenol

The conversion of *p*-nitrophenol to *p*-aminophenol was carried out in the presence of catalyst *C. papaya*-Mn-Zn NPs at room temperature 31 °C. Briefly, 10 mM of *p*-nitrophenol was made up into 100 mL in a flask using double distilled water. At the same time, 30 mg of sodium borohydride (NaBH_4_—reducing agent) was dissolved in double distilled water (5 mL). About 10 mL of *p*-nitrophenol and NaBH_4_ were mixed and stirred for 5 min. In addition, 1 mg of catalyst was added and continuously stirred for 5 min. The above reaction mixture was utilized and UV-Vis range was recorded at every 1 min time interval. Moreover, the same protocol was used for various catalyst weights, including 2 and 3 mg.

## 3. Results and Discussion

### 3.1. UV-Vis Spectra Analysis

**Figure 1:**
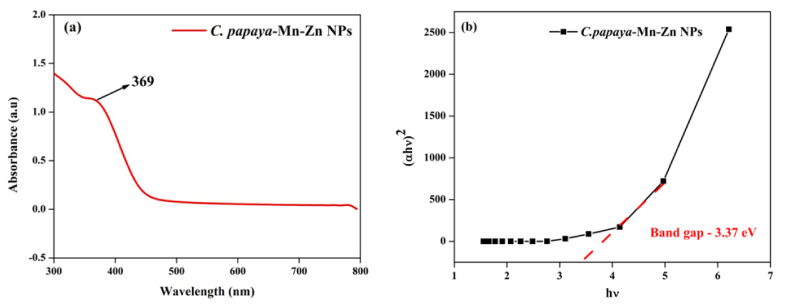
(**a**) UV-Vis analysis and (**b**) band gap of *C. papaya*-Mn-Zn NPs.

The maximum absorption was observed as 369 nm, which was due to the surface plasmon resonance (SPR), and it was analyzed using UV-Vis spectrophotometer, which confirmed the formation of bimetal oxide *C. papaya*-Mn-Zn NPs. The formation of nanoparticles was also confirmed by using the color of the solution. The color of the solution changed from green to dark brown. The bandgap of the nanoparticle was calculated from Tauc’s plot, which showed that the *C. papaya*-Mn-Zn NPs had a band gap value of 3.37 eV. Figure 1a,b show the λ max and band gap value of f. *papaya*-Mn-Zn NPs. Dhanalakshmi et al. synthesized Mn-doped ZnO NPs and the λ max value was observed as 360 nm due to a slight blue shift. This blue shift suggests the incorporation of Mn inside the ZnO lattice [27]. Mohammad et al. synthesized Mn-doped ZnO NPs using *Melastoma malabathricum* leaf extract and examined its optical properties, and its absorption maximum value was found to be 390 nm, which was due to red shift [28].

### 3.2. XRD Analysis

**Figure 2:**
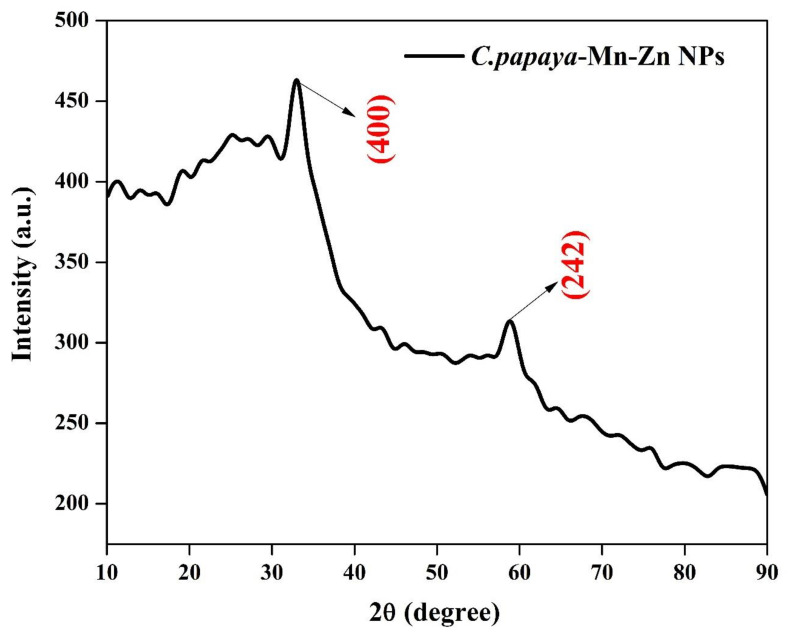
XRD pattern of *C. papaya*-Mn-Zn NPs.

The crystallite structure, size, and plane of the synthesized bimetal oxide nanoparticles were confirmed using XRD analysis. The nanoparticle formation was confirmed by comparing the peak with Joint Committee on Powder Diffraction Standards (JCPDS) card. From the XRD analysis, the peak observed at 2θ = 33.01 and 61.36° with respect to the plane (hkl) = (400) and (242) and these peaks are in agreement with the standard JCPDS card No. 65-5257. The synthesized nanoparticle has monoclinic structure, and the average crystallite size was found to as 22 nm with respect to the peaks 33.01 (400) and 61.36 (242). (Figure 2). Abdollahi et al. synthesized manganese-doped zinc oxide nanoparticles with different concentration. According to their report, no impurity peaks were attributed to manganese-related secondary phases, which confirmed the Mn atoms were located at substitutional sites [29]. Deepika et al. reported that no peaks appeared for manganese in spectra, which obviously showed that the lattice structure remains intact [30].

### 3.3. FT-IR Analysis

**Figure 3:**
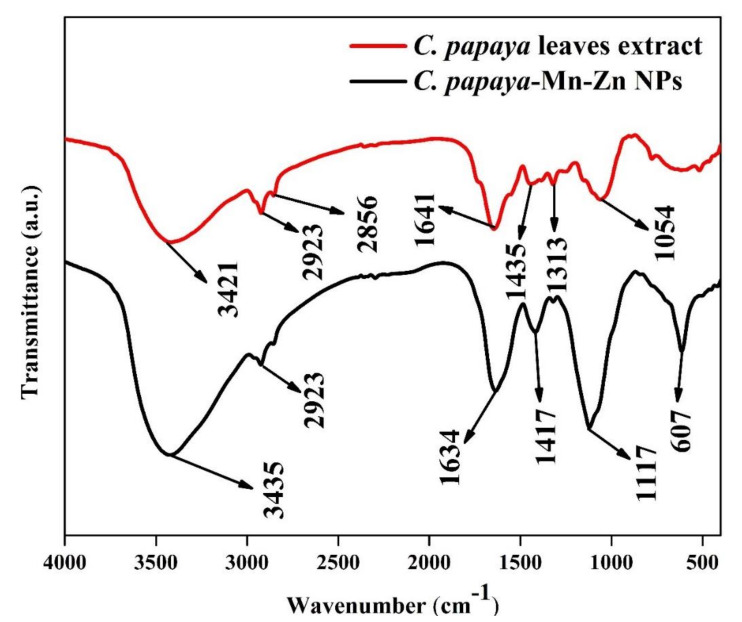
FT-IR analysis of *C. papaya*-Mn-Zn NPs.

The FT-IR spectrum depicted the presence of inorganic, organic constituents in the plant extract. Figure 3 shows the FT-IR analysis of *C. papaya* leaf extracts and *C. papaya*-Mn-Zn NP. The peak at 3421 cm^−1^ corresponds to the stretching vibrations of OH group for the fresh leaf extract [31]. The C–H stretching vibrations of the CH_3_ group appear at 2923 cm^−1^, while the peak at 2856 cm^−1^ corresponds to the symmetric stretching vibrations of CH_2_ group. The peak observed at about 1054 cm^−1^ corresponds to the C–O stretching, while the peak at 1634 cm^−1^ is allocated to the bending vibrations of the hydroxyl groups of the water molecule [31]. For the FTIR spectra of *C. papaya*-Mn-Zn NP, the band at 607 cm^−1^ corresponds to the Mn bending vibrations and the peaks at 1117 cm^−1^ and 1417 cm^−1^ correspond to the C–O–C bond and the carboxyl group asymmetric stretching, respectively [32]. The main difference in the FTIR spectra is the change in intensity of the characteristic peaks without a major shift in wavenumber, which indicates that there are only physical interactions due to the nanofiller interactions with the matrix and no covalent bond formation. 

### 3.4. SEM-EDX

**Figure 4:**
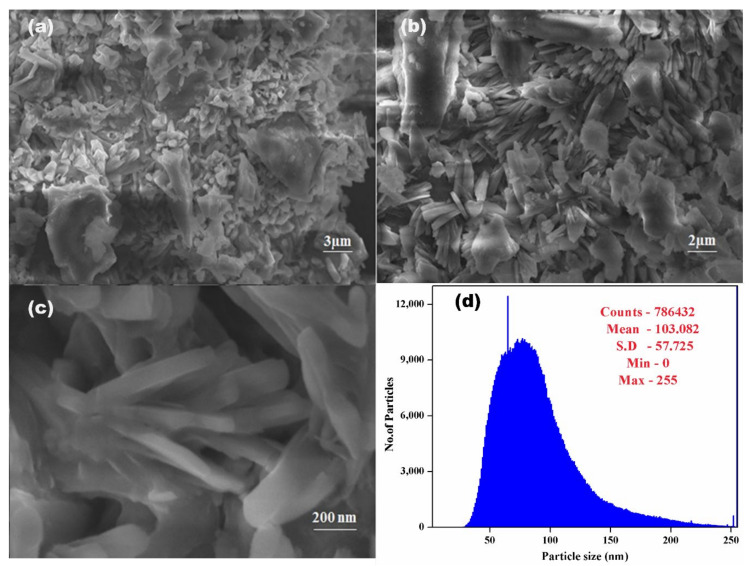
SEM images of *C. papaya*-Mn-Zn NPs at various magnifications (**a**) 3 µm, (**b**) 2 µm, (**c**) 200 nm and (**d**) particle size.

Figure 4 shows a scanning electron microscopy (SEM) of *C. papaya*-Mn-Zn NPs synthesized using the green approach. The synthesized *C. papaya*-Mn-Zn NPs shows nanosheet structure and as seen in Figure 4, very small particles coexist with massive aggregates. The nanosheet morphology of ZnO-doped Mn NPs was also observed by Maria et al. [33]. The average particle size of synthesized *C. papaya*-Mn-Zn NPs was found to be 103 nm. The borders of particles are well defined. The particles are joined in one or more locations. The absence of contrast in the particles indicates that the atoms of both metals are dispersed uniformly within them, resulting in the formation of Mn-Zn bimetal oxide particles. No discernible difference is seen in Figure 4, indicating that both metals are combined randomly during synthesis. 

**Figure 5:**
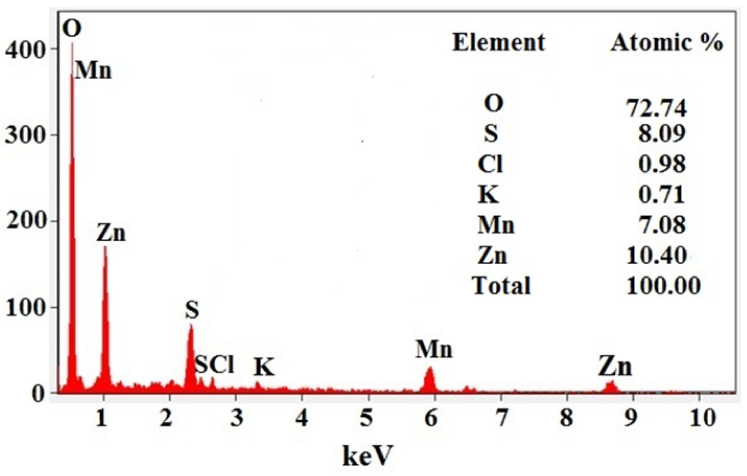
EDX analysis of *C. papaya*-Mn-Zn NPs.

The energy-dispersive X-ray diffraction (EDX) pattern of the synthesized *C. papaya*-Mn-Zn NPs is shown in Figure 5. The atomic and weight percentage of the *C. papaya*-Mn-Zn NPs were found out from EDX analysis. From the analysis, the atomic percentage was found to be 7.08 and 10.40 for Mn and Zn, respectively. The weight percent of Mn and Zn was found to be 15.23 and 26.63, respectively. In addition to the Mn and Zn, O was observed as a major peak which contains the atomic and weight% of 72.81 and 45.62, respectively. To this, sulfur, carbon, potassium was found as impurities in *C. papaya*-Mn-Zn NPs. EDX analysis not only used to find out the atomic and weight% but also to examine the chemical formation of the synthesized NPs. 

### 3.5. DLS Analysis

**Figure 6:**
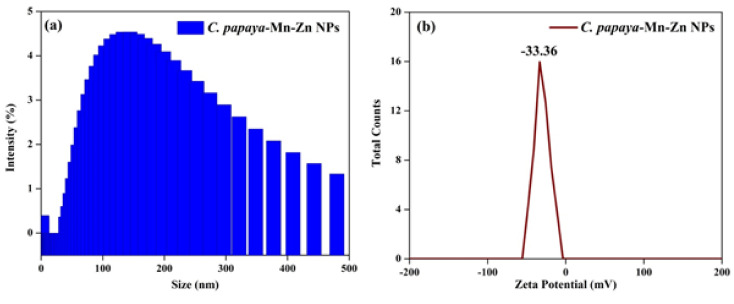
(**a**) Particle size and (**b**) Zeta potential of *C. papaya*-Mn-Zn NPs.

The hydrodynamic diameter of the synthesized *C. papaya-*Mn-Zn NPs was analyzed using a dynamic light scattering (DLS) particle size analyzer. The nanoparticle’s cumulative particle size and polydispersity index (PDI) was found to be 135.1 nm and 0.310, respectively. The PDI value suggests that the synthesized nanoparticle was homogeneously dispersed [34]. The surface charge of the synthesized *C. papaya-*Mn-Zn NPs was measured by zeta potential. Surface charge of the particle indicates the stability of synthesized *C. papaya-*Mn-Zn NPs. The result of zeta potential value for *C. papaya-*Mn-Zn NPs was around −33.36 mV at pH 7. The negative value of the zeta potential depicts the function of biomolecules on the surface of *C. papaya-*Mn-Zn NPs as capping agent. The nanoparticles have negative zeta potential value shows it generate repulsive forces amongst the particles, which are accountable for avoiding aggregation in the solution [35]. Figure 6a,b shows particle size and zeta potential of *C. papaya-*Mn-Zn NPs. 

### 3.6. Effect of pH on Optical Spectra and DLS

**Figure 7:**
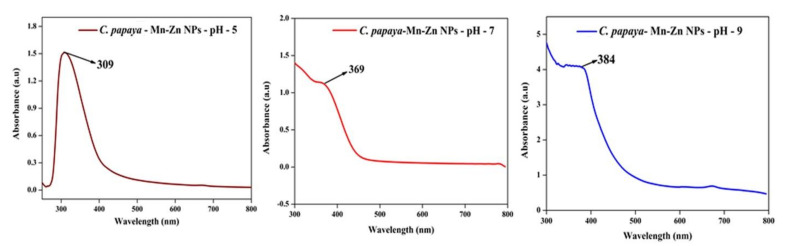
UV-Vis analysis of *C. papaya*-Mn-Zn NPs at different pH.

**Figure 8:**
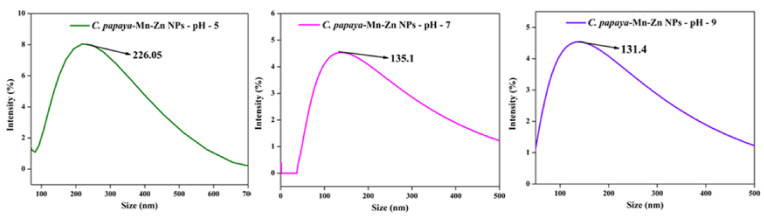
DLS analysis of *C. papaya*-Mn-Zn NPs at different pH.

At the alkaline pH range, the stability of cluster distribution and colloid formation was increased with a declined tendency for aggregation of the particles, due to complete charging of the clusters, which maximizes the repulsive electrostatic/electro steric interactions. A possible reason for this result was that during the elevated pH, the reaction rate was increased with subsequent crystallization into smaller particles, which involved the nucleation and growth processes of smaller particles. There are many factors that affect the size of nanoparticles. In this part, we addressed the impact of the pH on the optical spectra and DLS of *C. papaya*-Mn-Zn NPs by monitoring the size variation and optical spectra as a result of changing the pH of the solution. Absorption spectra at different values of the pH (5, 7, and 9) are shown in Figure 7. In acidic conditions, the absorption value decreases and size of the particle increases. The reason being the reducing agent instead of acting as a nucleating agent acts as a growth agent at low pH, hence leading to an increase in the size of the erstwhile particles. Hence, we presume that pH changes favor the controlling of particle size. The size of the nanoparticles was inversely proportional to pH and absorbance. Figure 8 shows the DLS analysis of *C. papaya*-Mn-Zn NPs at different pH.

### 3.7. Antioxidant Activity

#### 3.7.1. DPPH Scavenging Assay

**Figure 9:**
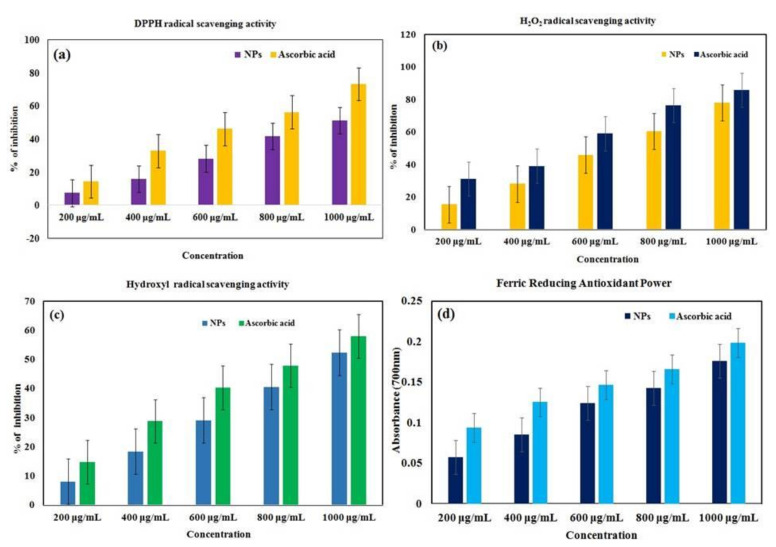
Antioxidant activity of *C. papaya*-Mn-Zn NPs with different assays: (**a**) DPPH, (**b**) H_2_O_2_, (**c**) Hydroxyl radical and (**d**) FRAP.

The DPPH scavenging experiment (Figure 9a) was conducted with the synthesized *C. papaya*-Mn-Zn NPs and standard ascorbic acid. The nanoparticle’s DPPH activity was shown to increase in dose-dependent manner. However, the synthesized *C. papaya*-Mn-Zn NPs inhibited DPPH scavenging activity at 1000 µg/mL concentration, and it was found to be effective up to 51.35%. The DPPH free radical scavenging experiment revealed that *C. papaya*-Mn-Zn NPs had a greater capacity for scavenging free radicals.

#### 3.7.2. Hydrogen Peroxide-Scavenging Assay

Greenly synthesized Mn-Zn NPs were capped with free radical scavenging phytoconstituents discovered in *C*. *papaya* leaf extract. The phenolic component of leaf extract can quickly donate an electron to hydrogen peroxide, neutralizing it in water [36]. The hydrogen peroxide scavenging activity of green-synthesized *C. papaya*-Mn-Zn NPs was studied at various doses range from 200 to 1000 µg/mL (Figure 9b). The present results indicated that *C. papaya*-Mn-Zn NPs had a higher scavenging activity (78.08%) at 1000 µg/mL, indicating that H_2_O_2_ action is likewise dose dependent.

#### 3.7.3. Hydroxyl-Radical Scavenging Activity

*C*. *papaya*-Mn-Zn NPs were synthesized by a green approach and capped with free radical scavenging phytoconstituents isolated from papaya leaf extract. The phenolic component of the leaf extract is capable of rapidly donating an electron to the hydroxyl radical, neutralizing it in the presence of water [37]. The hydroxyl radical scavenging activity of green-synthesized *C*. *Papaya*-Mn-Zn NPs was investigated (Figure 9c). The results revealed that *C*. *papaya*-Mn-Zn NPs (52.34%) had a greater scavenging capability at 1000 µg/mL, showing that hydroxyl radical action is also dose-dependent.

#### 3.7.4. Ferric-Reducing Antioxidant Power (FRAP) 

The antioxidant activity of synthesized *C*. *papaya*-Mn-Zn NPs was examined in this work, and it was discovered that *C*. *papaya*-Mn-Zn NPs are efficient antioxidants. The antioxidant activity of *C*. *papaya*-Mn-Zn NPs was assessed by their ability to reduce Fe^3+^ to Fe^2+^ ions which leads to the formation of ferrous-tripyridyltriazine complex. From Figure 9d, the reducing power of the catalyst was dose-dependent, which was found to be 0.17 at 1000 µg/mL. 

### 3.8. Antibacterial Activity

**Figure 10:**
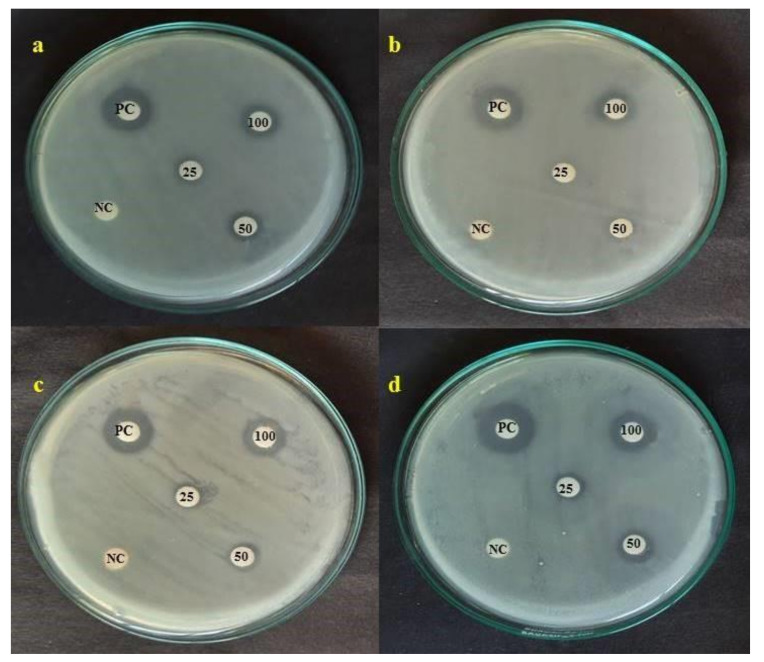
Antibacterial activity of *C. papaya*-Mn-Zn NPs against different pathogens: (**a**) *E. coli*; (**b**) *K. pneumonia*; (**c**) *S. aureus*; (**d**) *S. typhi*.

**Figure 11:**
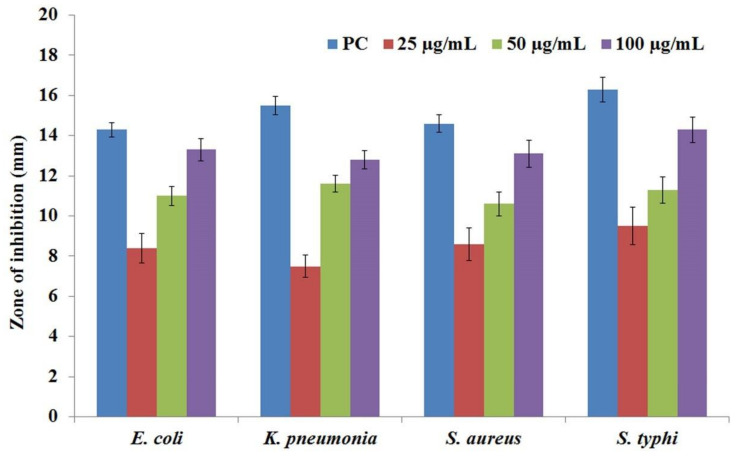
Zone of inhibition of *C. papaya*-Mn-Zn NPs against different pathogens.

The disc diffusion technique was used to assess the antibacterial activity of *C*. *papaya*-Mn-Zn NPs against *E. coli*, *K. pneumonia*, *S. aureus* and *S. typhi*. The extensive zone of inhibition surrounding the *C*. *papaya*-Mn-Zn NPs integrated discs established the antibacterial activity of *C*. *papaya*-Mn-Zn NPs and it has been shown in Figure 10. Among the tested concentrations, a maximum concentration of 100 µg/mL exhibited a higher zone of inhibition in *S. typhi* with 14.3 ± 0.64 mm, followed by *E. coli-*13.3 ± 0.55 mm, *S. aureus-*13.1 ± 0.69 mm and *K. pneumonia-*12.8 ± 0.47 mm. Figure 11 shows the zone of inhibition of *C. papaya*-Mn-Zn NPs against different pathogens. Antibacterial activity was fundamentally mediated by the interaction of the positively charged surface of NPs contrasts with the bacterial cell wall’s net negative charge [38]. The hydroxyl group attached to the surface of ZnO particles plays an important role in surface charge behavior. In addition to that, the protons at lower pH are likely transferred to the particle surface from the environment, thus causing a positive charge from surface ZnOH_2_ + groups. Under physiological conditions, the ZnO nanoparticles possess a strong positive surface charge [39]. In previous research, the electromagnetic interaction between bacteria and metal oxides has induced oxidation and cell death. The examined NPs exhibit bactericidal activity as a result of reactive oxygen species (ROS), notably hydroxyl radicals (OH), which cause phospholipid peroxidation and oxidative cell death [40].

### 3.9. Photodegradation Activity

**Figure 12:**
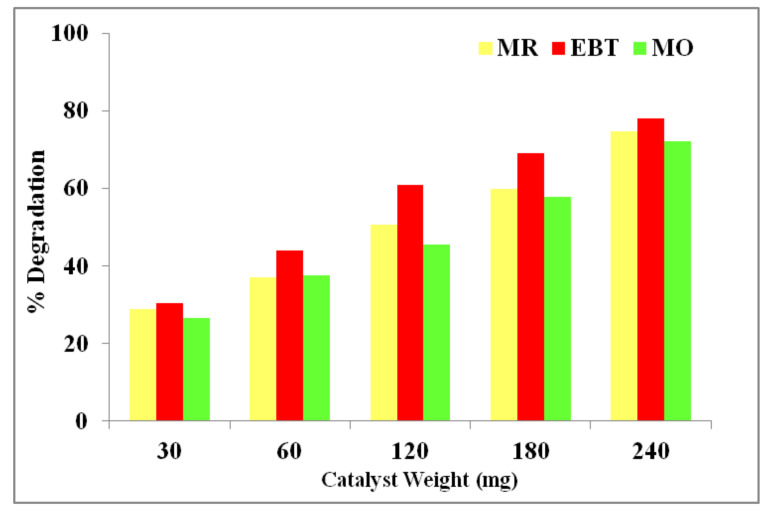
Plot of concentration vs. degradation percentage for dye degradation by *C. papaya*-Mn-Zn NPs.

The photocatalytic degradation efficiency of *C*. *papaya-*Mn-Zn NPs was studied against MR, EBT and MO as a pollutant with different photocatalyst weights (30, 60, 120, 180, and 240 mg) under solar irradiation with fixed concentration of dyes and pH of the dye was maintained to be 7 ± 0.1. In the presence of photocatalyst the decomposition of dyes was induced, and the reaction was carried up to 180 min. Figure 12 shows the degradation efficiency of *C. papaya-*Mn-Zn NPs with different catalyst weights. In the case of MR, the degradation efficiency was found to be 30.91, 39.25, 48.42, 62.70, and 78.54% with respect to 30, 60, 120, 180, and 240 mg, respectively. During the EBT degradation, the efficiency was found to be 34.00, 48.93, 64.45, 74.26, and 87.67% concerning 30, 60, 120, 180, and 240 mg, respectively. The degradation efficiency was found to be 46.14, 49.59, 58.77, 64.77, and 69.79% with respect to 30, 60, 120, 180, and 240 mg, respectively, during the MO degradation. The dye degradation process is primarily caused by the formation of electrons and holes on the catalyst surface during irradiation. These electrons and holes mix in a sequence of reactions to produce hydroxyl radicals. The hydroxyl radicals are reactive oxygen species that catalyze the degradation of the dye molecule into inorganic chemicals [41]. The degradation efficiency of the NPs was high possibly due to the large surface area which supplies more active sites to reactant molecules for removal of dye constituents from affected water. The increase in active sites leads to transfer and penetration of dye molecules to the adsorbent. The number of available active sites on the photocatalyst increases, and hence an increase in the number of hydroxyl radicals is produced, which can take part in the degradation of the dye solution. The dye solutions were degraded because of hydroxyl radicals. By increasing the weight of photocatalyst, the degradation efficiency remains constant because of excessive active sites present in the photocatalyst rather than dye molecules [42]. The possible mechanism of degradation of dye is illustrated below. The generation of electron–hole pairs is significant in dye degradation. The electron is excited to conduction band from valance band of the photocatalyst leaving behind a positive hole in a valance band. The oxygen molecule reacts with the electron in the conduction band to produce super oxide anions. The former super oxide anion reacts with water molecule to generate hydroxyl radical. The hydroxyl radical is formed during the interaction of electron–hole pairs with water and the formed hydroxyl radical plays a key role in destroying the dye constituents.

### 3.10. Catalytic Efficiency

**Figure 13:**
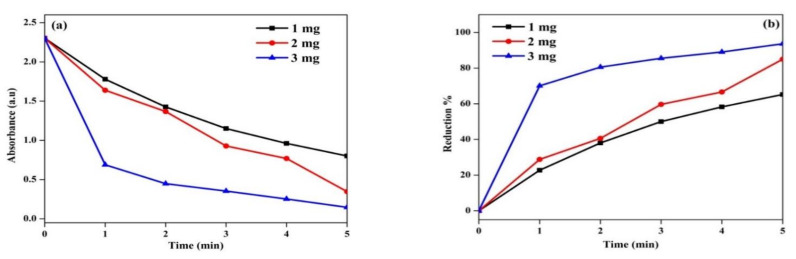
(**a**) Time vs. Absorbance. (**b**) Time vs. Reduction % of *C. papaya*-Mn-Zn NPs for *p*-nitrophenol reduction.

**Figure 14:**
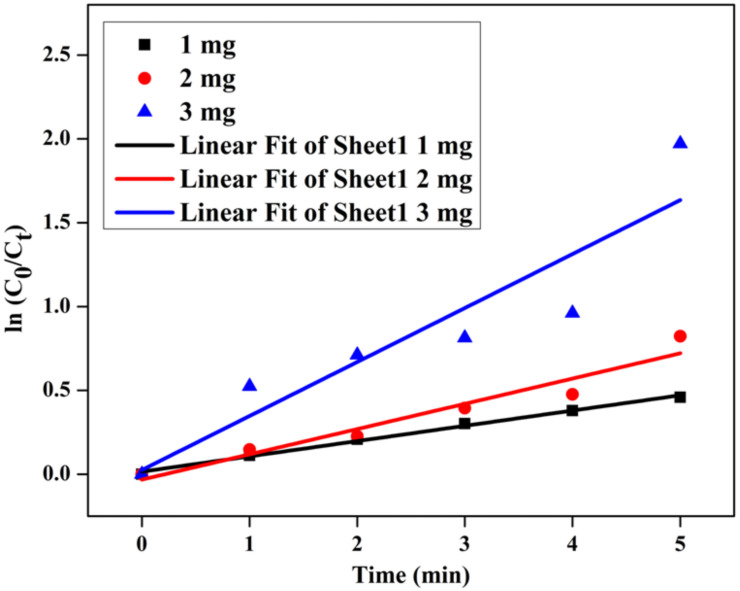
Time vs. ln (c_0_/c_t_) of *C. papaya*-Mn-Zn NPs for *p*-nitrophenol reduction.

The efficiency of the catalyst was evaluated to reduce 4-nitrophenol as a pollutant. The pollutant *p*-nitrophenol reduction was confirmed using *C. papaya-*Mn-Zn NPs, and absorbance and % reduction was shown in Figure 13a,b, respectively. The conversion reaction was carried out at 31 °C (room temperature), and the reduction reaction’s results confirmed that *p*-nitrophenol was converted to *p*-aminophenol. During reduction reaction, the phenolate ion was formed as an intermediate, and it was changed to less toxic *p*-aminophenol. A peak which appeared at 400 nm denoted the *p*-nitrophenol, and after 5 min, the peak noticed at 300 nm confirmed the formation of *p*-aminophenol. The different catalyst concentrations including 1, 2 and 3 mg showed reductions up to 65.21%, 84.97% and 93.63%, respectively. The linear regression co-efficient was shown in Figure 14. Moreover, as the dose of catalyst increased, the reduction process was found to increase, and the concentration of *p*-nitrophenol decreased. It was due to more active sites available in the catalyst for the reduction in *p*-nitrophenol [43].

## 4. Conclusions

In summary, a facile, economically viable, and green approach for the synthesis of Mn-Zn bimetal oxide NPs were developed by employing *C. papaya* leaves extract as a reducing as well as capping agent. The synthesized NPs were found to be 103 nm, spherical in shape, with an agglomerated structure. The photocatalytic efficacy of synthesized nanoparticles was confirmed using the degradation of methyl red, Erichrome black T, and methyl orange dyes. However, it also exhibited antioxidant properties, which qualifies them for future pharmacological investigation. As a consequence, Erichrome black T (87.67%), methyl red dye (78.54%), and methyl orange dye (69.79%) deteriorated. Mn-Zn nanoparticles are utilized as a catalyst for *p*-nitrophenol reduction. The results of the study indicated that *C*. *papaya* leaf extract may be used to synthesize *C*. *papaya*-Mn-Zn NPs. Most importantly, *C*. *papaya*-Mn-Zn NPs might be utilized to degrade dyes in wastewater. In addition, it demonstrated the bioactivity profile of *C*. *papaya*-Mn-Zn NPs in antioxidant and antibacterial properties.

## Data Availability

Not applicable.
